# Supplementary material for: Enhancement of Vancomycin Potential against Pathogenic Bacterial Strains via Gold Nano-Formulations: A Nano-Antibiotic Approach
Source: Materials (Basel). 2022 Jan 31;15(3):1108. doi: 10.3390/ma15031108 (PMC8840600; doi:10.3390/ma15031108)
Supplement: Supplementary file 1 [file materials-15-01108-s001.zip › materials-1561404-supplementary.pdf]

materials

MDPI

Article

Enhancement of Vancomycin Potential against Pathogenic Bacterial Strains via Gold Nano-Formulations: A Nano-Antibiotic Approach

Turki Al Hagbani [1], Hemant Yadav [2], Afrasim Moin [1], Amr Selim Abu Lila [1,3], Khalid Mehmood [4], Farhan Alshammari [1], Salman Khan [5], El-Sayed Khafagy [6,7], Talib Hussain [8,*], Syed Mohd Danish Rizvi [1,*] and Marwa H. Abdallah [1,3]

1 Department of Pharmaceutics, College of Pharmacy, University of Ha'il, Ha'il 81442, Saudi Arabia; t.alhagbani@uoh.edu.sa (T.A.H.); afrasimmoin@yahoo.co.in (A.M.); a.abulila@uoh.edu.sa (A.S.A.L.); frh.alshammari@uoh.edu.sa (F.A.); mh.abdallah@uoh.edu.sa (M.H.A.)
2 Department of Pharmaceutics, RAK College of Pharmaceutical Sciences, RAK Medical & Health Sciences University, Ras Al Khaimah 11172, United Arab Emirates; hemant@rakmhsu.ac.ae
3 Department of Pharmaceutics and Industrial Pharmacy, Faculty of Pharmacy, Zagazig University, Zagazig 44519, Egypt
4 Department of Pharmacy, Abbottabad University of Science and Technology, Havelian 22010, Pakistan; adckhalid@gmail.com
5 Nanomedicine and Nanotechnology Lab, Department of Biosciences, Integral University, Lucknow 226026, India; salmank@iul.ac.in
6 Department of Pharmaceutics, College of Pharmacy, Prince Sattam Bin Abdulaziz University, Al-kharj 11942, Saudi Arabia; e.khafagy@psau.edu.sa
7 Department of Pharmaceutics and Industrial Pharmacy, Faculty of Pharmacy, Suez Canal University, Ismailia 41522, Egypt
8 Department of Pharmacology and Toxicology, College of Pharmacy, University of Ha'il, Ha'il 81442, Saudi Arabia
* Correspondence: mdth_ah@yahoo.com (T.H.); syeddanishpharmacy@gmail.com (S.M.D.R.)

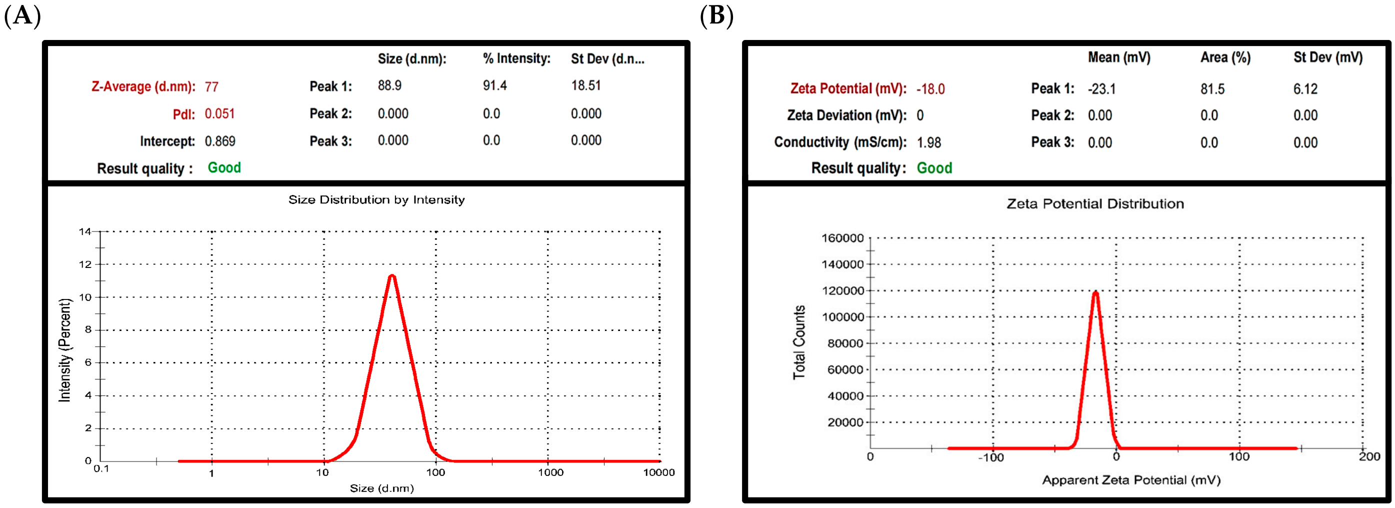

Figure S1. (**A**) DLS, (**B**) Zeta-potential of V-GNPs.
